# Twenty-Four Hour Total and Dietary Fat Oxidation in Lean, Obese and Reduced-Obese Adults with and without a Bout of Exercise

**DOI:** 10.1371/journal.pone.0094181

**Published:** 2014-04-08

**Authors:** Audrey Bergouignan, Elizabeth H. Kealey, Stacy L. Schmidt, Matthew R. Jackman, Daniel H. Bessesen

**Affiliations:** 1 Anschutz Health & Wellness Center at the University of Colorado, Anschutz Medical Campus, Aurora, Colorado, United States of America; 2 Division of Endocrinology, Metabolism, and Diabetes, University of Colorado, School of Medicine, Aurora, Colorado, United States of America; 3 Denver Health Medical Center, Denver, Colorado, United States of America; INSERM/UMR 1048, France

## Abstract

**Background:**

It has been hypothesized that obese and reduced-obese individuals have decreased oxidative capacity, which contributes to weight gain and regain. Recent data have challenged this concept.

**Objective:**

To determine (1) whether total and dietary fat oxidation are decreased in obese and reduced-obese adults compared to lean but increase in response to an acute exercise bout and (2) whether regular physical activity attenuates these metabolic alterations.

**Design:**

We measured 24-hr total (whole-room calorimetry) and dietary fat (^14^C-oleate) oxidation in Sedentary Lean (BMI = 21.5±1.6; n = 10), Sedentary Obese (BMI = 33.6±2.5; n = 9), Sedentary Reduced-Obese (RED-SED; BMI = 26.9±3.7; n = 7) and in Physically Active Reduced-Obese (RED-EX; BMI = 27.3±2.8; n = 12) men and women with or without an acute exercise bout where energy expended during exercise was not replaced.

**Results:**

Although Red-SED and Red-EX had a similar level of fatness, aerobic capacity and metabolic profiles were better in Red-EX only compared to Obese subjects. No significant between-group differences were seen in 24-hr respiratory quotient (RQ, Lean: 0.831±0.044, Obese: 0.852±0.023, Red-SED: 0.864±0.037, Red-EX: 0.842±0.039), total and dietary fat oxidation. A single bout of exercise increased total (+27.8%, p<0.0001) and dietary (+6.6%, p = 0.048) fat oxidation across groups. Although exercise did not impact RQ during the day, it decreased RQ during sleep (p = 0.01) in all groups. Red-EX oxidized more fat overnight than Red-SED subjects under both resting (p = 0.036) and negative energy balance (p = 0.003) conditions, even after adjustment for fat-free mass.

**Conclusion:**

Obese and reduced-obese individuals oxidize as much fat as lean both under eucaloric and negative energy balance conditions, which does not support the hypothesis of reduced oxidative capacity in these groups. Reduced-obese individuals who exercise regularly have markers of metabolic health similar to those seen in lean adults. Both the acute and chronic effects of exercise were primarily observed at night suggesting an important role of sleep in the regulation of lipid metabolism.

## Introduction

Although a number of effective weight loss strategies are available [1]–[3], the major challenge in obesity treatment is in preventing lost weight from being regained. Weight lost over 3–6 months is often regained in 1–3 years, with over half of the lost weight being regained over the first 6 months [4], [5]. The factors responsible for weight regain are not clear.

A number of mechanisms have been proposed including changes in metabolism, appetite and energy expenditure favoring fat storage, food intake, and positive energy balance respectively [6]. There is a widely held view that a defect in fat oxidation [7] due to reduced mitochondrial function [8], [9] or content [10]–[14] is present in obese adults and may predispose to weight gain and regain [15]. Some studies however do not support this idea [10]–[12], [14]. The use of different methods to measure fat oxidation may explain these divergent results. Most studies have assessed total fat oxidation by calorimetry whereas measures of dietary fat oxidation using tracer methods may provide additional insights. Studies in rodent models of obesity have consistently shown that there is a defect in dietary fat oxidation in pre-obese and obese rats that is even more marked in reduced-obese animals [6], [16]–[18]. A number of investigators have performed dietary fat tracer studies in humans [19]–[25] but few in obese individuals [23], [26], and only one in reduced-obese [22]. Free fatty acids (FFA) or dietary fat predominate in distinct metabolic states. FFAs are high in a fasting state while triglycerides (TG) originating from dietary fat predominate following a meal. By tracking the metabolic fate of dietary fat, we hoped to better understand the alterations in fat metabolism that promote weight regain following weight loss in order to develop better strategies for long-term weight maintenance.

Individuals who successfully maintain weight loss over the long-term report high levels of physical activity [27], [28]. There is evidence suggesting that physical activity counters the biological adaptations to weight loss that facilitate weight regain [6]. Exercise increases fat oxidation [29] and dietary fat is an important source of fat fuel during or after exercise in lean individuals [30]–[32]. The acute effect of exercise or of habitual physical activity on dietary fat metabolism in obese or reduced-obese individuals is currently unknown.

We tested the hypothesis that obese and reduced-obese individuals have lower 24-hr total and dietary fat oxidation compared to lean healthy male and female adults; this defect being more pronounced in reduced-obese individuals. We determined the effect of an acute bout of moderate-intensity exercise on 24-hr total and dietary fat oxidation. We additionally compared fat oxidation in previously obese individuals who lost weight with diet only to those who lost weight with the combination of diet and exercise in order to examine the effect of regular physical activity on the regulation of fat metabolism. Exercise could alter fat balance by producing a state of negative energy balance or through changes in fat oxidation produced by the exercise *per se*. To allow effects from either of these mechanisms to be tested we did not replace energy burned in the exercise bout in these studies.

## Material and Methods

### Institutional Approval

The study was approved by the Colorado Multiple Institutional Review Board and the Scientific Advisory Board of the Clinical Translational Research Center (CTRC) at the University of Colorado-School of Medicine (UCSOM). Informed written consent was obtained from each subject. Subjects were studied between 2009 and 2011.

### Subjects

Healthy Lean (body mass index (BMI)  =  19–25 kg/m^2^), Obese (BMI  =  30–40 kg/m^2^) and reduced-obese (BMI  =  25–40 kg/m^2^) adults (19–45 years) were recruited by email and flyers. Reduced-obese subjects were required to have lost a minimum of 10% of their body weight and maintained the reduced weight for at least 6 months prior to being studied. Subjects were excluded if they had a history of cardiovascular disease, diabetes, thyroid disease, renal dysfunction, smoked, used medications affecting weight or energy balance or had a change in body weight of >5% in the 6 months prior to being studied. Female volunteers were excluded if they planned to become pregnant, were lactating, were less than 6 months from delivery or were post-menopausal. Lean and Obese individuals were all sedentary (excluded if they self-reported >1 exercise bout/week or moderate/vigorous physical activity >1 hr/wk). Reduced-obese subjects were divided into two groups, individuals who self-reported having lost and maintained weight through caloric restriction alone and were sedentary (Red-SED, sedentary as defined for lean and obese subjects) and a second group of individuals who reduced their weight and maintained it by both dieting and exercising (Red-EX; self-reported moderate intensity exercise at least 3 times per week for at least 30 min per session). Volunteers who passed the initial screening were invited to participate in a health history and physical examination. BMI was confirmed by measuring height and weight while wearing only socks, undergarments, and a hospital gown. Resting metabolic rate (RMR) was measured by hood indirect calorimetry (ParvoMedics Model: TrueOne 2400, Sandy, UT) and body composition by DXA (Hologic Discovery-W, Bedford, MA) as described previously [33]. The characteristics of the 38 subjects included in this study are presented in Table 1.

**Table 1:** Subjects characteristics and fasting hormone and metabolites concentration.

	Lean	Obese	Red-SED	Red-EX
n (Male/Female)	10 (5M/5F)	9 (4M/5F)	7 (3M/4F)	12 (6M/6F)
**Age**	29.9±6.8	34.8±6.5	34.3±8.8	33.3±7.7
**Weight (kg)**	64.0±10.0	98.3±10.3^a^	73.7±10.0^b^	81.9±9.8 ^a,b^
**BMI (kg/m^2^)**	21.5±1.6	33.6±2.5 ^a^	26.9±3.7 ^a,b^	27.3±2.8 ^a,b^
**FFM (kg)**	48.9±8.9	59.5±10.6 ^a^	50.8±7.4 ^b^	57.6±10.7 ^a^
**FM (kg)**	14.1±4.1	36.2±6.1 ^a^	21.9±5.7 ^a,b^	23.5±7.2 ^a,b^
**% BF (%)**	22.5±6.2	38.1±7.3 ^a^	30.0±6.1 ^a,b^	29.0±9.1
**RMR (kcal)**	1476±197	1754±244 ^a^	1513±184 ^b^	1689±245 ^a^
**RMR_adjFFM_ (kcal)**	1600±78	1655±163	1560±65	1637±123
**VO_2peak_ (mL/min)**	1839±438	1578±427	1456±466	2084±874
**VO_2peak/FFM_ (mL/kg/min)**	37.4±3.6	25.6±4.6 ^a^	27.7±6.2 ^a^	34.8±10.0 ^b^
**Average of daily steps**	7763±1674	7511±2598	8718±3140	9129±2629
**High level activity (IPAQ)**	677±498	1127±1139 ^a^	440±630 ^a,b^	2226±1051 ^a,b,c^
**HOMA**	2.36±1.05	5.19±4.01^a^	3.70±0.65^a^	2.40±1.11^b,c^
**Glucose (mg/dl)**	81.2±5.2	92.9±7.0^a^	88.3±5.9^a^	85.7±9.2
**Insulin (μUI/ml)**	13.0±2.8	26.0±12.9^a^	17.0±3.2^a^	11.3±5.0^b,c^
**FFA (μEq/l)**	577.3±5.2	533.9±163.7	502.0±144.7	479.5±147.5
**Triglycerides (mg/dl)**	77.3±25.8	152.0± 68.5^a^	99.6±43.7	70.0±12.5^b^
**Cholesterol (mg/dl)**	160.3±33.2	171.7±24.1	170.3±32.6	139.1±16.6^b,c^
**Total HDL (mg/dl)**	55.9±14.1	42.7±13.08^a^	46.5±15.0	47.7±9.7

One-Way ANOVA: ^a^ P<0.05 vs Lean group; ^b^ P<0.05 vs Obese group, ^c^ P<0.05 vs RED-SED group BMI, Body mass index; FFA, free fatty acids; FFM, Fat free mass: FM, Fat mass; RED-EX, Reduced-Exercise group; RED-SED, Reduced-Sedentary group; RMR, resting metabolic rate; RMR_adjFFM_, resting metabolic rate adjusted for fat free mass; VO2_peak_, maximal aerobic capacity; VO2peak/FFM, maximal aerobic capacity normalized by fat free mass. Values are mean ± SD

### Aerobic capacity and habitual physical activity

To estimate aerobic capacity, we determined the maximal aerobic capacity (VO_2peak_) of each subject using a progressive exercise test on an electronically braked stationary cycle ergometer. VO_2peak_ was determined from the average of the highest three 30-s measurements. To be accepted as valid, the test was required to meet two of the following three criteria: *1*) a RQ >1.1, *2*) heart rate within 10 beats/min of 85% of age-predicted maximum, and 3) an increase in oxygen uptake (VO_2_) in response to the final workload of <2.0 ml·kg^−1^·min^−1^. To assess habitual physical activity, subjects filled the International Physical Activity Questionnaire and wore a pedometer (Digi-Walker, New-Lifestyles, Inc. Lee's Summit, MO) for one week prior to beginning studies. On a separate occasion following the initial exercise test a second test was done to more precisely define the level of exercise intensity on the cycle ergometer that was equal to 60% of their previously determined VO_2peak_. This intensity was then used during the exercise condition of the study.

### Study design

Each subject completed two trials performed at the CTRC at the UCSOM in random order separated by one month: one under resting conditions and one that included an acute exercise bout. Both tests were performed in the follicular phase in women. Between the two tests, subjects were instructed to maintain their regular physical activity, i.e. sedentary activities for Lean, Obese and Red-SED groups and habitual levels of physical activity for the Red-EX group. Prior to each inpatient stay in the room calorimeter, subjects consumed a controlled outpatient diet (15% protein, 30% fat, 55% carbohydrate) to stabilize energy and macronutrient intake during a 3d baseline period. During these 3 days, the Lean, Obese and Red-SED groups were instructed to maintain their typical sedentary lifestyle during the outpatient phase while the Red-EX group was asked to not engage in any structured physical activity. During the exercise visit, subjects completed a moderate-intensity exercise bout on a cycle ergometer at 60% of VO_2peak_ at 4pm for a period of time that resulted in the expenditure of an amount of energy equal to 15% of 24 hr energy expenditure (EE). The duration of the exercise ranged from 30 to 57 minutes. Lean, Obese, Red-SED and Red-EX subjects exercised on average 40.3±1.2, 44.7±1.8, 48.1±2.7 and 42.6±2.1 minutes, respectively. The calories expended during the exercise were not replaced, which placed the subjects in an energy deficit of ≈15% compared to the resting condition.

### Study diet

The energy requirements for the “run-in diet” period and the chamber stays were calculated based on an estimate of resting metabolic rate (RMR) derived from the average of 1) direct measurement by hood indirect calorimetry (ParvoMedics Model: TrueOne 2400, Sandy, UT) and 2) an estimate using the following equation: [(23.9 x FFM in kg) + 372], where fat-free mass (FFM) was measured by DXA (Hologic Discovery-W, Bedford, MA). RMR was then multiplied by an activity factor (1.4–1.65) based on the average number of steps taken by subjects during the week of baseline pedometer monitoring. This level of dietary energy content was provided during all run-in and chamber stays in both study conditions. Specifically, during the exercise visit, subjects received the same amount of food that they received in the resting conditions. All meals were prepared by the CTRC metabolic kitchen and had the same macronutrient composition (15% protein, 30% fat, 55% carbohydrate). Subjects were required to consume all food provided and no food other than non-caloric beverages, included caffeine beverages, was permitted. Two optional food modules (200 kcal each, macronutrient composition matched the diet) were provided in the event that the subject experienced hunger. To verify compliance to the diets, subjects were required to return empty food containers to the CTRC metabolic kitchen.

### Whole-room calorimeter protocol

**Figure 1:**
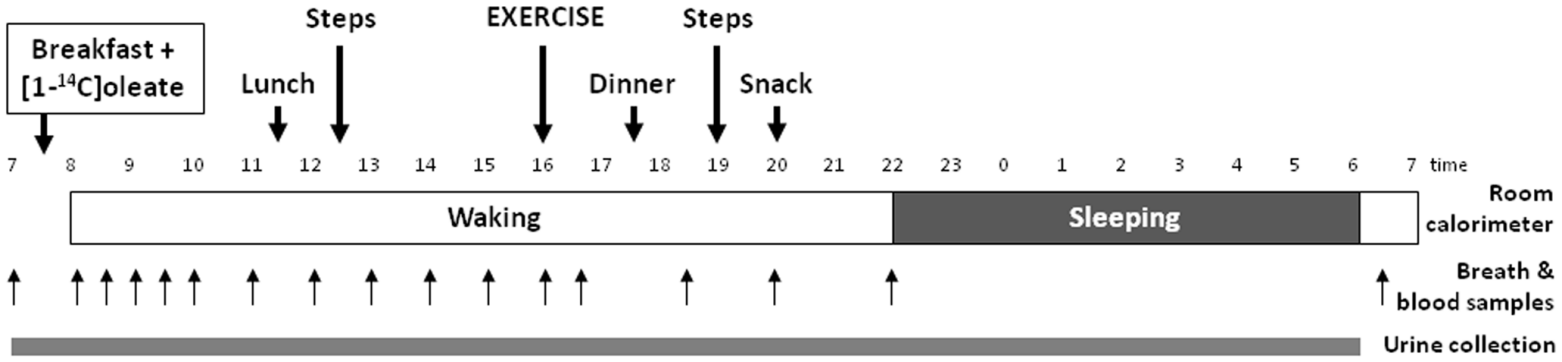
Protocol of the tests in the room calorimeter.

The study protocol is depicted in Figure 1. After collecting baseline breath, urine and arterialized venous blood samples, a liquid breakfast (Boost High Protein) was served to subjects (25% energy intake; 55% carbohydrates, 15% protein, and 30% fat) to which 20 μCi of [1-^14^C] oleate (MORAVEK, Brea, CA) had been added. Subjects entered the room calorimeter at 0800 and exited at 0700 the following day. Two 20-min bouts of bench-stepping exercise were performed at 72 steps/min at 1230 and 1900 to mimic free-living physical activity. Subjects were free to move about the calorimeter during other times of the day, but primarily this time was spent in sedentary activities (reading, computer use, watching TV). Subjects were instructed to remain awake, and to go to bed at 2200 during each calorimeter stay and to wake up the following day at 0600. Lunch and dinner were provided at 1130 and 1730 respectively (each 30% daily energy), and a light snack (15% daily energy) was provided at 2000. Venous blood samples were obtained every 30 min for the first 2hrs after breakfast was consumed and then every hour for 6hrs, and subsequently after the exercise bout (or 1645 on the resting day), 1830, 2000, 2200 and the following day at 0700. On the exercise day, exercise started at 4pm for a duration ranged from 30 to 57 min depending on the time/intensity needed to expend the required energy. To obtain blood samples, subjects extended their arm through an air-tight port in the calorimeter wall. Breath samples were collected at these same time points. Twenty-three hour urine samples were collected to estimate protein disappearance.

### 24-hr EE and substrate oxidation

Twenty-three hour data on respiratory gas exchange were extrapolated to 24-hr values. Twenty-four hour EE and total substrate oxidation were determined from oxygen consumption (VO_2_), carbon dioxide production (VCO_2_) and nitrogen excretion in the urine, as previously described [33].

To determine waking and nighttime respiratory quotient (RQ), we averaged the minute-to-minute VCO_2_ and VO_2_ values during waking and sleeping time periods. Waking and sleeping periods corresponded to the exact time when each subject fell asleep and woke up. Bedtime was set at 2200 and waking time at 0600 the following day. Because sleep fat oxidation was not an *a priori* outcome of the study, nitrogen excretion was measured in a single 24-hr collection. As a result, for purposes of estimating waking and sleeping fat oxidation, it was assumed that urinary nitrogen excretion was constant over 24-hrs.

### Dietary fat oxidation

Expired CO_2_ was collected in 3.0-ml aliquots of a 2∶1 mixture of methanol and methylbenzethonium (hyamine) hydroxide (Sigma). The ^14^C content of these samples was then measured by scintillation counting (LS6500, Beckman Coulter, Brea, CA). Background activity, determined by counting a sample containing only scintillation fluid and hyamine hydroxide, was subtracted from experimental values. Disintegrations/min were converted to μCi/MCO_2_ expired per hour over 24hrs, and then the specific activity of ^14^ C/g fat in the test meal was used to convert this value to gram of dietary fat oxidized.

### Hormones and metabolites

Whole blood was added to a preservative (3.6 mg EDTA plus 2.4 mg glutathione in distilled water). Serum was separated after spinning and stored at −80°C until analyzed. Samples were assayed for TG and FFA by using commercial enzymatic assays using the Beckman - Olympus AU400e Chemistry Analyzer (Brea, CA).

### Statistical analysis

Data were expressed as means ± SD unless otherwise stated and analyzed with SPSS software (version 21.0). Data were analyzed under resting condition by a one-way ANOVA to determine group differences. Then, data were analyzed by using a repeated-measures analysis of variance with test as the repeated measure and group as main effect, followed by a post-hoc Tukey test. In some cases, ANCOVA was used to examine differences that remained after adjusting for relevant covariates [energy balance, energy intake, fat mass, FFM, gender]. Pearson correlation coefficients were calculated to examine the relationships between parameters of interest. Statistical significance was assumed when p was <0.05 and <0.1 for main effects and interaction, respectively.

## Results

### Anthropometric, aerobic and metabolic characteristics

**Figure 2:**
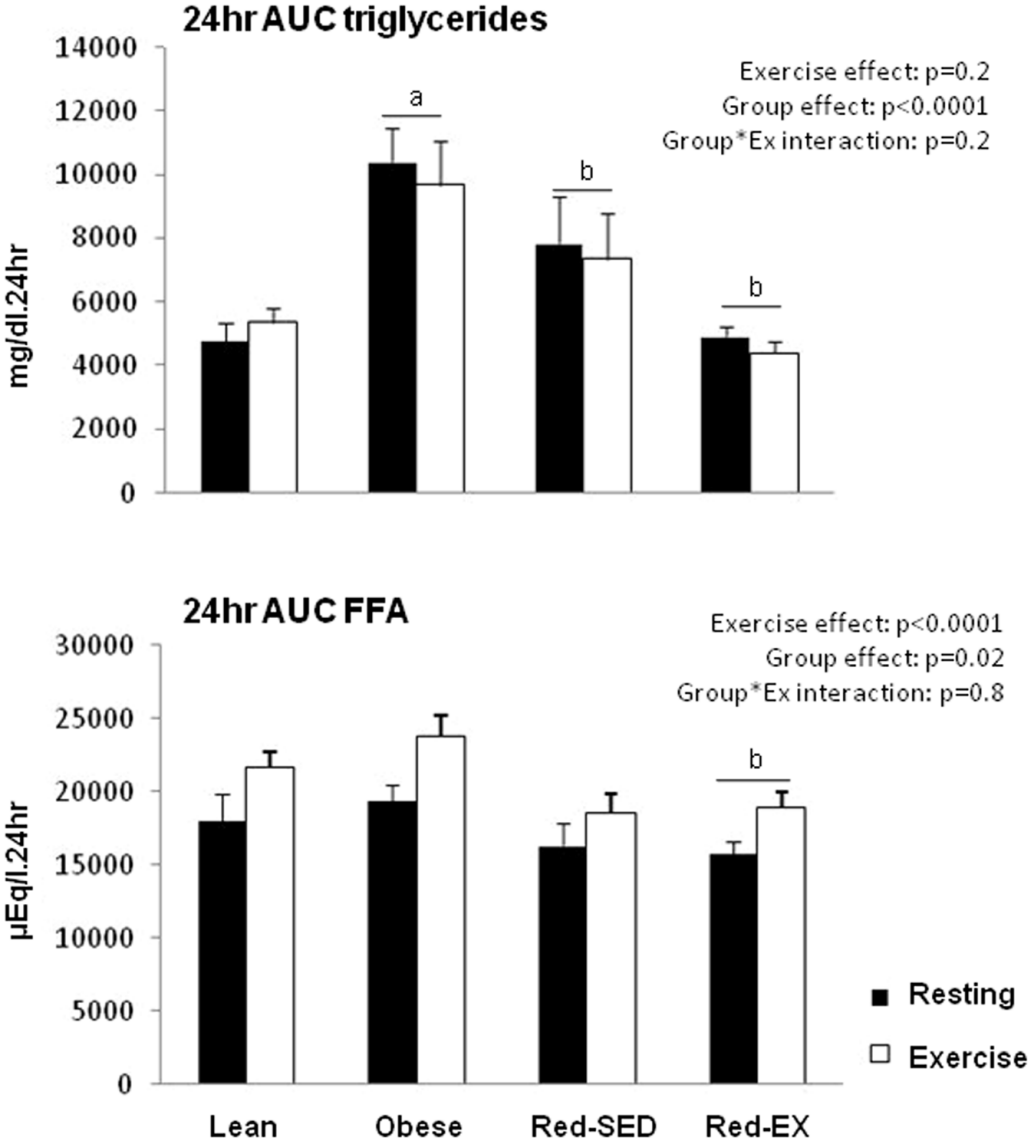
Cumulative responses of plasma triglycerides (top) and free fatty acids (FFA; bottom) concentrations expressed as the area under the curve (AUC) over 24 h in lean (n = 10), obese (n = 9), sedentary reduced-obese (Red-SED, n = 7) and exercising reduced-obese (Red-Ex, n = 12). Statistics are presented on the Figure.

Subject characteristics are shown in Table 1 and 24-hr area under the curve (AUC) of TG and FFA in Figure 2. Obese subjects had higher body weights, BMI, FFM, fat mass (FM), and percentage of body fat (%BF) than Lean subjects (p<0.05 for all) and had a 32% lower aerobic capacity per kg of FFM (p = 0.005). They had a less healthy metabolic profile as indicated by the higher values of HOMA (p<0.0001), fasting glucose (p = 0.022), insulin (p = 0.001), TG (p = 0.003) and 24-hr AUC TG (p<0.0001), and lower fasting HDL concentrations (p = 0.049).

Red-SED subjects reported very low levels of high-intensity activity (less than 600MET-min/week), which, based on the IPAQ scoring criteria [34], placed them in the “inactive individuals” category. This level was lower than those reported by both Lean and Obese subjects (p<0.05 for both). With levels of high-intensity activity comprised between 600 and 1500MET-min per week, these two groups are considered moderately active. Red-SED had lower body weight, BMI, FFM, FM and %BF than Obese subjects (p<0.05 for all), but their aerobic capacity was not different. Despite their lower fatness, their metabolic characteristics were similar to those seen in the Obese group.

The Red-EX subjects self-reported very high levels of intense physical activity (above 1500 MET-min/week) as compared to the three other groups (p<0.05 for all) and had a tendency to walk more as measured by a pedometer. Based on the IPAQ scoring they would be categorized as people exceeding the public health recommendations for physical activity. Their aerobic capacity was as high as that of Lean subjects, higher than that of Obese (p = 0.032) and tended to be higher than that of Red-SED (p = 0.09) subjects when normalized for FFM. Although the Red-EX group had similar body fat level as compared to the Red-SED group, they had a much healthier metabolic profile. Compared to the Obese group, the TG levels of the Red-EX group were markedly lower (fasting TG: p<0.0001; 24-hr AUC TG: p<0.0001). Furthermore, their insulin sensitivity (HOMA, p = 0.050), fasting insulin (p = 0.049) and total cholesterol (p = 0.040) concentrations were lower than those measured in the Red-SED group.

BMI was positively correlated with HOMA (r^2^ = 0.434, p<0.0001), fasting insulin (r^2^ = 0.266, p<0.0001) and TG (r^2^ = 0.215, p = 0.004), 24-hr AUC insulin (r^2^ = 0.439, p<0.0001) and TG (r^2^ = 0.279, p = 0.001). Similar positive relationships were observed between %BF or FM and these metabolic parameters. On the contrary, aerobic capacity normalized for FFM was negatively correlated with HOMA (r^2^ = 0.124, p = 0.038), fasting insulin (r^2^ = 0.151, p = 0.017) and TG (r^2^ = 0.152, p = 0.017), 24-hr AUC insulin (r^2^ = 0.120, p = 0.036) and TG (r^2^ = 0.181, p = 0.008) and fasting total cholesterol (r^2^ = 0.229, p = 0.003).

### Energy expenditure and balance

Both RMR (Table 1; p<0.05) and 24-hr EE (Table 2; p = 0.005) were higher in Obese and Red-EX as compared to Lean and Red-SED groups. These differences disappeared when adjusted for FFM. On average, the total energy cost of the exercise, i.e. energy expended during the exercise bout and after as the excess post-exercise oxygen consumption (EPOC), was 318.0±115.4, 340.0±109.4, 365.7±98.8 and 361.2±123.6 kcal for the Lean, Obese, Red-SED and Red-EX subjects, respectively. No statistical differences were noted. Across the groups, the exercise bout increased 24-hr EE by 15.6% (p<0.0001) as expected, and negatively impacted energy balance (p<0.0001). While energy balance was not statistically different from zero in any group under resting conditions (unpaired t-test with energy balance equal zero), the Red-EX group had a significantly more positive energy balance than the Obese group (84.1±161.2 vs. -107.3±153.6 kcal respectively, p = 0.012; Table 2). Because of these group-differences, we statistically adjusted the outcomes presented below for energy balance.

**Table 2:** Room calorimeter results over 24

	Lean		Obese		Red-SED		Red-EX		RM-ANOVA, p values		
	Resting	Exercise	Resting	Exercise	Resting	Exercise	Resting	Exercise	Group	Test	Group*Test
n	10		9		7		12				
**Energy intake (kcal)**	2005±278	2004.7±279	2376±286^a^	2376±286^a^	2080± 269^b^	2094±282	2371±358^a^	2371±358^a^	.019	NS	NS
**Energy expenditure (kcal)**	2035±251	2353±280	2483±162^a^	2823±215^a^	2033±289^b^	2399±317	2287±334^a^	2648±389^a^	.005	<.0001	NS
**Energy balance (kcal)**	−30.7±45.6	−348.7±85.2	−107.3±153.6	−447.3±139.3	46.6±138.7	−305.5±153.5	84.1±161.2^b^	−277.1±193.2	.024	<.0001	NS
**Cost of exercise (kcal)**		318.0±115.4		340.0±109.4		365.7±98.8		361.2±123.6			
**RQ**	0.831±0.044	0.821±0.049	0.852±0.023	0.832±0.032	0.864±0.037	0.863±0.041	0.842±0.039	0.836±0.026	NS	NS	NS
***Macronutrient intake (g)***											
**Fat**	68.5±9.5	68.5±9.5	81.0±9.4^a^	81.0±9.4^a^	71.4±9.0	71.3±9.1	80.4±11.7^a^	80.4±11.7^a^	.019	NS	NS
**Carbohydrate**	263.3±36.9	262.7±37.8	314.3±39.3^a^	314.3±39.3^a^	274.6±40.2	274.6±40.2	312.0±47.3^a^	312.0±47.3^a^	.019	NS	NS
**Protein**	76.9±10.1	76.9±10.1	90.8±10.5^a^	90.8±10.5^a^	80.5±10.5	80.6±10.4	90.5±13.5^a^	90.5±13.5^a^	.021	NS	NS
***Macronutrient oxidation (g)***											
**Fat**	104.2±37.4	129.6±47.0	104.5±25.3	143.3±35.7	75.7±33.8	97.0±45.0^b^	104.9±37.8	129.7±38.7	NS	<.0001	NS
**Carbohydrate**	218.2±76.1	228.5±105.7	308.9±51.2^a^	301.0±74.2^a^	264.8±59.1	322.2±78.0	254.5±67.3	286.5±56.2	.03	NS	NS
**Protein**	49.4±16.6	57.4±15.6	72.9±25.0	72.6±13.5	71.5±17.0	58.8±5.7	74.2±22.3^a^	74.8±19.9^a^	.029	NS	.069
***Macronutrient balance (g)***											
**Fat**	−35.6±34.3	−61.1±46.8	−23.5±25.8	−62.2±30.8	−4.2±29.4	−25.7±42.8	−24.5±34.3	−49.3±34.6	NS	<.0001	NS
**Carbohydrate**	45.1±74.4	34.3±92.8	5.4±48.1	13.4±89.8	9.8±69.6	−47.6±72.4	57.5±73.9	25.5±54.2^c^	NS	NS	NS
**Protein**	27.5±18.0	19.5±14.8	17.8±24.0	18.2±10.5	9.0±11.2^a^	21.8±8.6	16.3±13.3	15.7±14.1	NS	NS	.068

Tukey post-hoc: ^a^ P<0.05 vs. Lean group, ^b^ P<0.05 vs. Obese group, ^c^ P<0.05 vs. RED-SED group.

RED-EX, Reduced-Exercise group; RED-SED, Reduced-Sedentary group; RQ, respiratory quotient.

Values are mean ± SD.

### Twenty-four hour total substrate oxidation and balance

**Figure 3:**
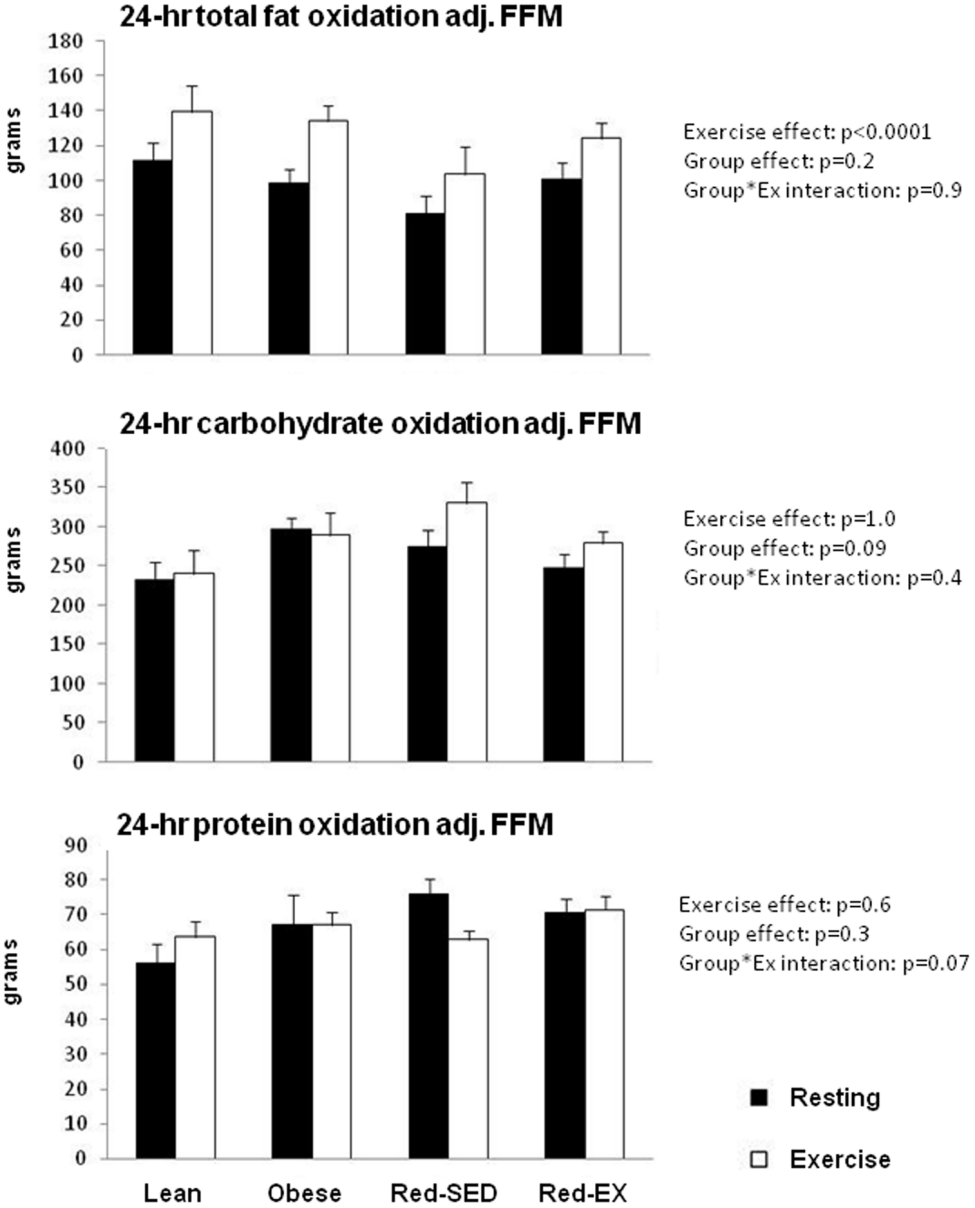
Twenty four hour total fat (top), carbohydrate (middle) and protein (bottom) oxidation in lean (n = 10), obese (n = 9), sedentary reduced-obese (Red-SED, n = 7) and exercising reduced-obese (Red-Ex, n = 12) after adjustment for differences in fat-free mass (FFM). Statistics are presented on the Figure.

Under resting conditions, no significant between-group differences were noted in both 24-hr RQ and total fat oxidation (Table 2). Twenty-four hour carbohydrate oxidation was significantly higher in the Obese as compared to the Lean group (p = 0.039, Table 2) and the Lean group oxidized less protein than the Obese (p = 0.074) and Red-EX (p = 0.028) groups. Similar results were observed when adjusting for gender. These differences in both carbohydrate and protein oxidation however were no longer present after adjusting for FFM (Figure 3).

Acute exercise did not modify 24-hr carbohydrate oxidation, but significantly increased 24-hr fat oxidation by 28% on average across the four groups (p<0.0001, Table 2). This effect of exercise on 24-hr fat oxidation however disappeared when energy balance was added as a covariate, suggesting that this effect was primarily due to the energy deficit. Although the acute exercise did not alter protein oxidation in Lean, Obese and Red-EX groups, Red-SED subjects oxidized less protein over 24-hr under exercise compared to resting conditions (−12.8±14.0 g, interaction: p = 0.069), even when energy balance was taken into account (interaction: p = 0.081). These results remained unchanged when adjusted for FFM (Figure 3), gender or when expressing substrate oxidation as percentage of 24hr EE (data not shown). Similar effects of exercise on nutrient balance were seen.

### Waking and sleeping RQ and substrate oxidation

**Figure 4:**
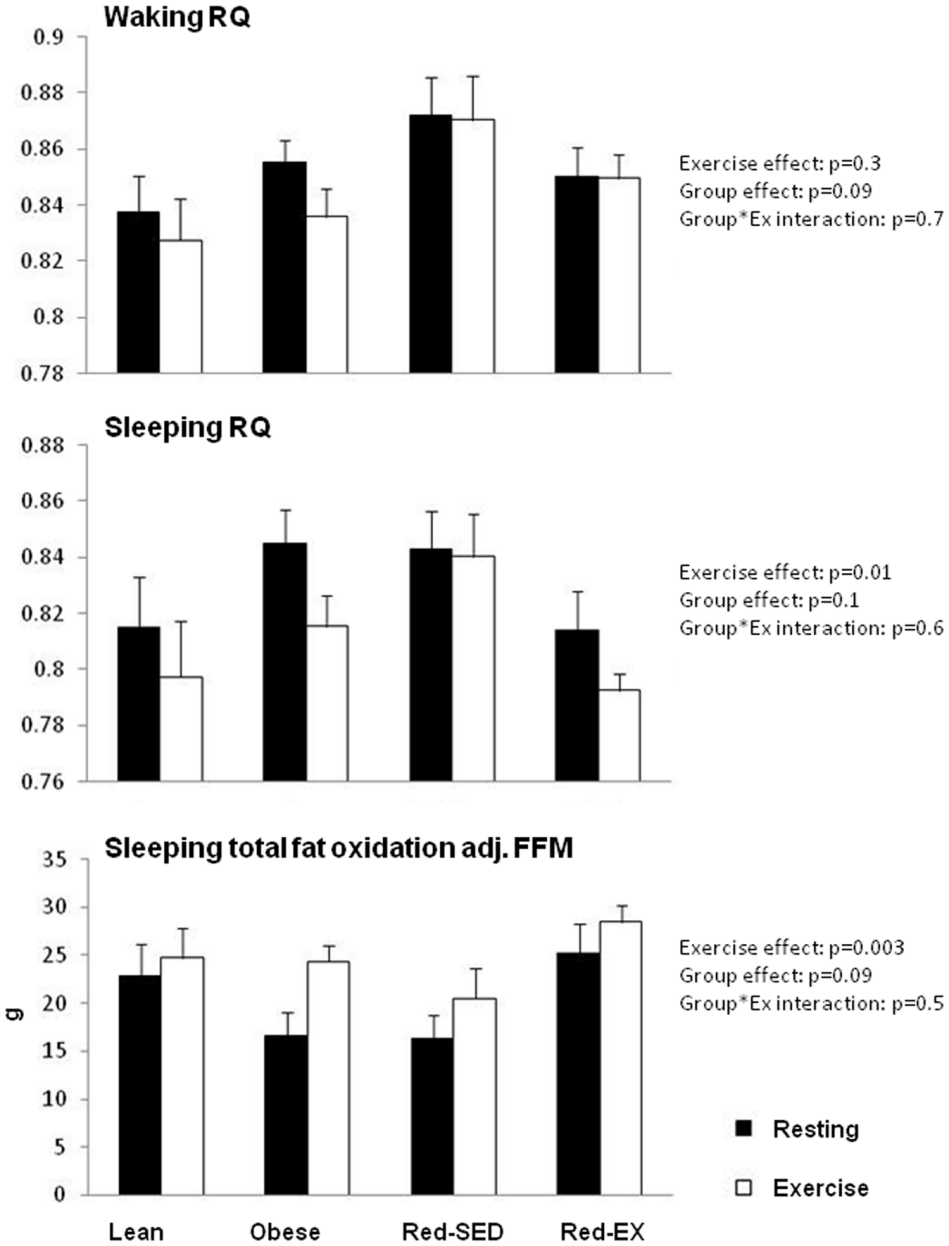
Respiratory quotient during waking (top) and sleeping (middle) periods and total fat oxidation during sleep in lean (n = 10), obese (n = 9), sedentary reduced-obese (Red-SED, n = 7) and exercising reduced-obese (Red-Ex, n = 12). Statistics are presented on the Figure.

In Figure 4, we distinguished RQ between waking and sleeping periods. Over the waking time period, EE significantly increased from 1698±257 to 2036±291 kcal in response to the acute exercise (p<0.0001, data not shown) but RQ was not altered. During sleep, however, RQ significantly decreased (p = 0.013) in the four groups while EE remained unchanged (resting conditions: 521±76 kcal, exercise conditions: 531±75 kcal; data not shown). Across all groups we further observed that during sleep fat oxidation increased from 20.9±10.0 to 25.0±8.8 g (p = 0.056) following a day with an exercise bout while carbohydrate oxidation remained unchanged (resting conditions: 40.5±17.6 vs. exercise conditions: 35.4±17.1 g, p = 0.097). Unexpectedly, Red-EX subjects oxidized 67% more total fat overnight than Red-SED subjects (25.8±10.7 vs. 15.5±6.9 g respectively, p = 0.036) following a resting day and 56% more (29.7±6.8 vs. 19.0±9.1 g, p = 0.009) following a day that included an exercise bout. These differences tended to persist after adjusting for FFM and considering FM, FFM, gender, energy intake and balance as covariates (group effect: p = 0.09; Tukey post-hoc under resting conditions: p = 0.065 and exercise conditions: p = 0.033), suggesting the existence of metabolic differences during sleep between these two groups. No such between-group differences were observed over the waking period.

### Twenty-four hour dietary fat oxidation

**Figure 5:**
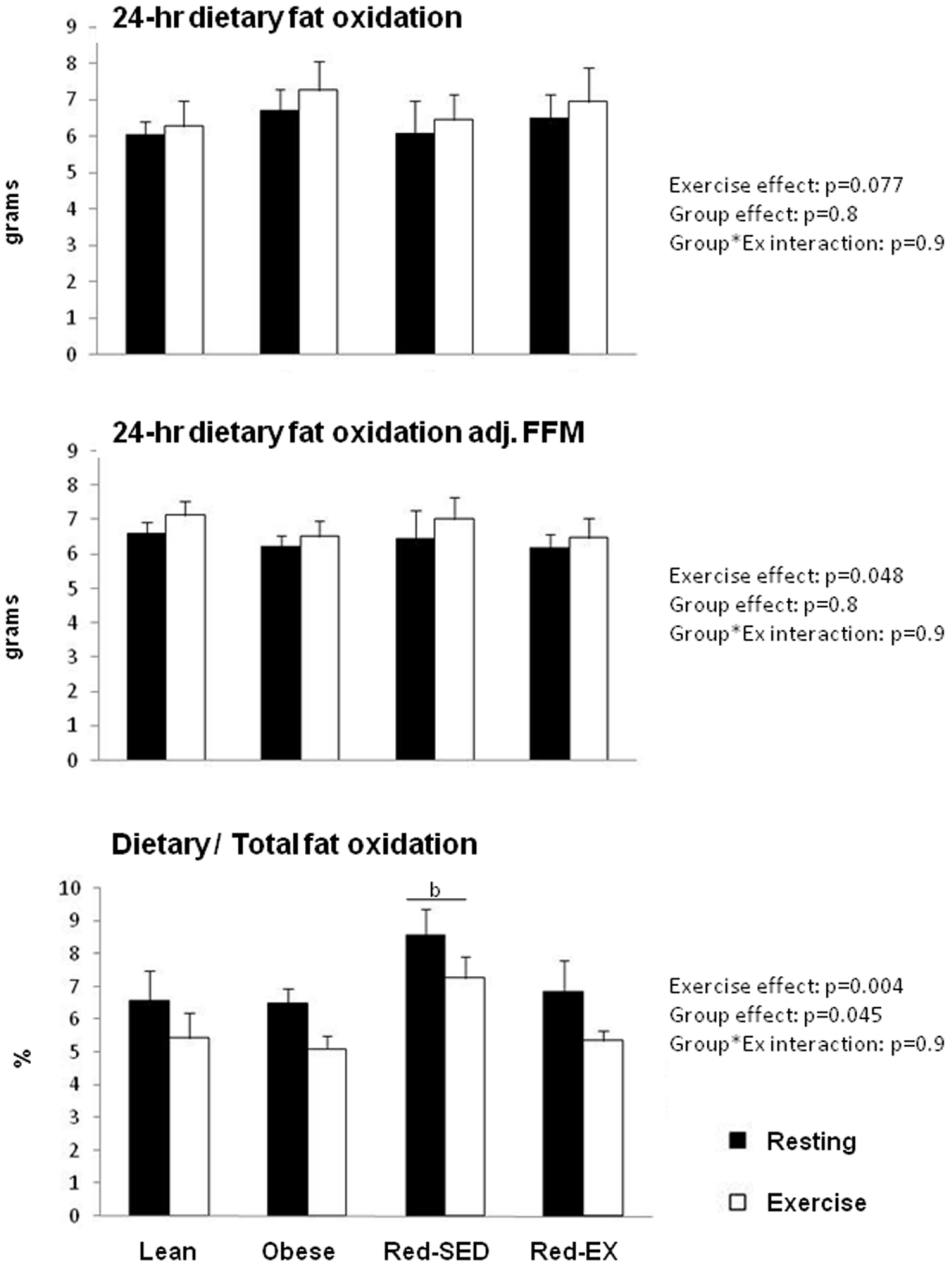
Dietary fat oxidation, i.e. the amount of fat oxidized over 24-hr coming from the breakfast meal only before (top) and after adjustment for fat-free mass (FFM) (middle), and the ratio of total and dietary fat oxidation (bottom) in lean (n = 10), obese (n = 9), sedentary reduced-obese (Red-SED, n = 7) and exercising reduced-obese (Red-Ex, n = 12). Statistics are presented on the Figure.

Under resting conditions, 24-hr oxidation of a dietary fat tracer consumed as part of the breakfast meal did not differ between the groups (Figure 5). Exercise tended to increase 24-hr oxidation of dietary fat by 6.6% across all groups (p = 0.077). This effect became significant after adjustment for FFM (p = 0.048). Exercise also significantly decreased the contribution of dietary fat from the breakfast to total 24-hr fat oxidation (p = 0.004). By taking the two tests together, a group effect was observed (p = 0.045) with Red-SED subjects using a greater proportion of fat coming from breakfast than Obese subjects (7.9±2.0% vs. 5.8±1.5%, p = 0.051).

### Twenty-four hour plasma FFA and triglycerides concentrations

While a single bout of exercise significantly increased 24-hr AUC FFA (p<0.0001) it did not alter the 24-hr AUC TG (Figure 2). Although all subjects responded similarly to exercise, the Obese group had a higher 24-hr TG AUC than Lean (p<0.0001), Red-SED (p = 0.054) or Red-EX (p<0.0001) and a higher 24-hr FFA AUC than Red-EX (p = 0.032) and Red-SED (p = 0.078) subjects. Twenty-four hour AUC FFA positively correlated with 24-hr fat oxidation under both resting (r^2^ = 0.116, p = 0.036) and exercise (r^2^ = 0.157, p = 0.014) conditions.

## Discussion

Contrary to our hypothesis, obese and reduced-obese individuals oxidized as much dietary and total fat as lean individuals over 24-hr. While sedentary reduced-obese did not have a healthier metabolic profile than obese subjects, weight-reduced subjects who exercised regularly had a level of aerobic fitness and a metabolic profile that were similar to that observed in lean individuals. Regular exercise likely plays a key role in improving metabolic health towards levels observed in lean healthy persons.

We showed that when compared to lean control subjects, obese subjects have similar 24-hr RQ profiles and fat oxidation rates and also increase 24-hr total fat oxidation in response to an acute bout of exercise to a similar degree. In a previous study, we had also observed no difference between obese and lean subjects in 24-hr RQ, fat oxidation and muscle oxidative capacity in response to a dietary intervention (low-fat vs. high-fat isocaloric diet) [35]. While studies examining short-term responses to metabolic challenges have demonstrated reduced fat oxidation in obese individuals [36]–[38], our observations that over 24-hr obese subjects can increase fat oxidation in response to a high-fat diet or a bout of exercise does not support the idea of a mitochondrial defect in fat oxidation in these people. Obese subjects also oxidized as much dietary fat as lean subjects. Previous studies [24] had observed lower dietary fat oxidation in obese compared to lean individuals when measured with deuterated palmitate over 12hrs. The discrepancy may be explained by differences in the metabolic fate of the fatty acids used (oleate vs. palmitate) [39], in the labeling itself (^2^H vs. ^14^C) or in the duration of the measurements (12hrs vs. 24hrs). It is also important to note that we, like others [23], [24], used a single-meal design limiting the assessment of dietary fat oxidation to only fat consumed with breakfast. By assessing dietary fat oxidation from three different tracers mixed into breakfast, lunch and dinner, a recent study [20] showed greater dietary fat oxidation in obese compared to lean adults. Further investigations are required to better understand the role of dietary fat in the regulation of body weight.

Previous studies in weight-reduced humans and rodents [6] found a preferential reliance upon carbohydrate for energy needs in association with reduced oxidation of both dietary fat [40] and postprandial total fat [22]. We however observed no difference in either 24-hr dietary or total fat oxidation between reduced-obese, obese and lean subjects. While these studies examined the metabolic adaptations to weight-reduced state immediately at the end of a weight loss program creating energy deficit [22], we studied individuals who lost weight but were weight stable for at least 6 months. Our findings may thus characterize the metabolic response to a chronically weight-reduced state while the previous studies may have observed changes in response to negative energy balance. The lower 24-hr AUC TG and the greater proportion of dietary fat being oxidized in reduced-obese subjects compared to obese however suggest differences in trafficking of dietary fat likely characterized by a greater proportion of dietary fat being used for immediate energy needs in weight-reduced individuals. The between-group differences in FFA concentrations and the correlation observed between 24-hr FFA concentrations and total fat oxidation further suggests that the inter-individual variability in total fat oxidation may primarily be due to differences in endogenous rather than exogenous fat oxidation. Further studies need to better delineate the dietary and plasma fat trafficking in obese and reduced-obese states to better understand the metabolic adaptations to weight-reduction.

We unexpectedly showed that acute exercise increased fat oxidation over 24-hr but the time of day during which exercise increased the ratio of fat to carbohydrate oxidation was not during waking when exercise increased EE but during sleep. Previous studies [33], [41] also have shown that changes in 24-hr substrate oxidation in response to exercise were associated with differences in RQ during sleep. In these studies [33], [41] exercise was performed in lean normal-weight sedentary or trained subjects in the morning and/or in the evening at either 45% or 55% VO_2_peak and for a duration that aimed at increasing 24hr EE by 20% or up to 1.8*RMR. Regardless of the time of the exercise (morning, afternoon or evening), the intensity, the energy cost of the exercise, or the training status and adiposity of the subjects, the nocturnal metabolic response to daytime exercise seems to be a consistant phenomenon. We further observed that if no differences were noted in 24-hr fat oxidation between sedentary and active weight-reduced individuals, the subjects who exercised regularly used more fat during sleep than their sedentary counterparts. Altogether our results suggest that the beneficial effects of both acute and chronic exercise on fat metabolism primarily occur during sleep. As has been noted in longitudinal and cross-sectional studies, we further observed that reduced-obese subjects who exercised regularly had higher levels of aerobic fitness [42], [43], insulin sensitivity [43], [44], HDL cholesterol [42], and lower fasting and 24-hr TG levels as compared to sedentary reduced-obese individuals, despite similar level of body fat. Aerobic fitness was also favorably associated with insulin sensitivity and lipemia, which provides additional evidence supporting the benefits of exercise in mitigating the adverse effects of unhealthy weight.

Some limitations exist. The decision to not replace calories expended in the acute bout of exercise does not allow us to distinguish the effect of exercise from that of negative energy balance. This choice was based on evidence showing that subjects do not spontaneously increase their food intake within 24-hr in response to exercise or if they do they do not completely match EE in exercise and are still in energy deficit (49). Future studies could determine which of these factors is most important. Another limitation is the assumption that protein oxidation during the day and night were the same. Before we performed the study we did not foresee the importance of the nighttime period in the metabolic response to exercise and so did not collect day and night urine samples separately. Finally the underlying mechanisms, i.e. dietary and plasma fat trafficking in plasma and muscle, changes in muscle glycogen stores and synthesis in response to exercise, and nutrient disposal, are lacking and will need to be investigated in future studies.

In conclusion, the fact that both obese and reduced-obese individuals have similar fat oxidation rates and ability to increase fat oxidation in response to acute exercise than lean subjects does not support the idea that mitochondrial dysfunction is a root cause of altered fat metabolism. The obese state is however associated with unhealthy metabolic patterns that are not improved by weight loss induced by dieting only. Although exercise training is known to have a modest effect on weight loss itself, the prescription of exercise in addition of dieting seems to be necessary to restore metabolism towards a pre-obese state. Interestingly the effects of both chronic and acute exercise were detected primarily during sleep suggesting that sleep may play a key role in maintaining homeostasis. Understanding the regulation of lipid metabolism during sleep warrants future studies.
